# Proton Radiobiology

**DOI:** 10.3390/cancers7010353

**Published:** 2015-02-12

**Authors:** Francesco Tommasino, Marco Durante

**Affiliations:** 1 GSI Helmholtzzentrum für Schwerionenforschung, Department of Biophysics, Darmstadt 64291, Germany; E-Mail: f.tommasino@gsi.de; 2 Technische Universität Darmstadt, Institut für Festkörperphysik, Darmstadt 64291, Germany

**Keywords:** proton therapy, radiobiology, RBE, biological range, target fragmentation

## Abstract

In addition to the physical advantages (Bragg peak), the use of charged particles in cancer therapy can be associated with distinct biological effects compared to X-rays. While heavy ions (densely ionizing radiation) are known to have an energy- and charge-dependent increased Relative Biological Effectiveness (RBE), protons should not be very different from sparsely ionizing photons. A slightly increased biological effectiveness is taken into account in proton treatment planning by assuming a fixed RBE of 1.1 for the whole radiation field. However, data emerging from recent studies suggest that, for several end points of clinical relevance, the biological response is differentially modulated by protons compared to photons. In parallel, research in the field of medical physics highlighted how variations in RBE that are currently neglected might actually result in deposition of significant doses in healthy organs. This seems to be relevant in particular for normal tissues in the entrance region and for organs at risk close behind the tumor. All these aspects will be considered and discussed in this review, highlighting how a re-discussion of the role of a variable RBE in proton therapy might be well-timed.

## 1. Introduction

Nowadays proton therapy (PT) represents an established alternative to photon radiotherapy for the treatment of specific types of cancer. According to the latest updates of the Particle Therapy Co-Operative Group (PTCOG), 36 PT centers are currently in operation and this number is going to increase in the next few years [1]. Starting in the 1950s, over 100,000 patients have already undergone PT treatments. Traditionally, the therapeutic use of proton beams (and of charged particles in general) is motivated primarily by their inverted depth-dose profile compared to photons, being characterized by the so-called Bragg peak. Thus, the damage induced in healthy tissues surrounding the tumor is limited. Several beams of different energies can then be combined in order to achieve the prescribed dose in a region as large as the target volume, resulting in the production of what is defined as the Spread-Out Bragg Peak (SOBP). Consequently, deep-seated tumors, especially those close to Organs At Risk (OAR), represent an ideal configuration for exploiting the advantageous physical characteristics of charged particle beams. The reduced dose in healthy tissues is particularly important for the treatment of pediatric patients, given their risk of developing late morbidity, including secondary cancers [2]. As a general rule, the integral dose delivered to the patient will be 2–3 times lower with protons when compared to X-rays delivered by Intensity Modulated Radiation Therapy (IMRT) [3].

**Figure 1:**
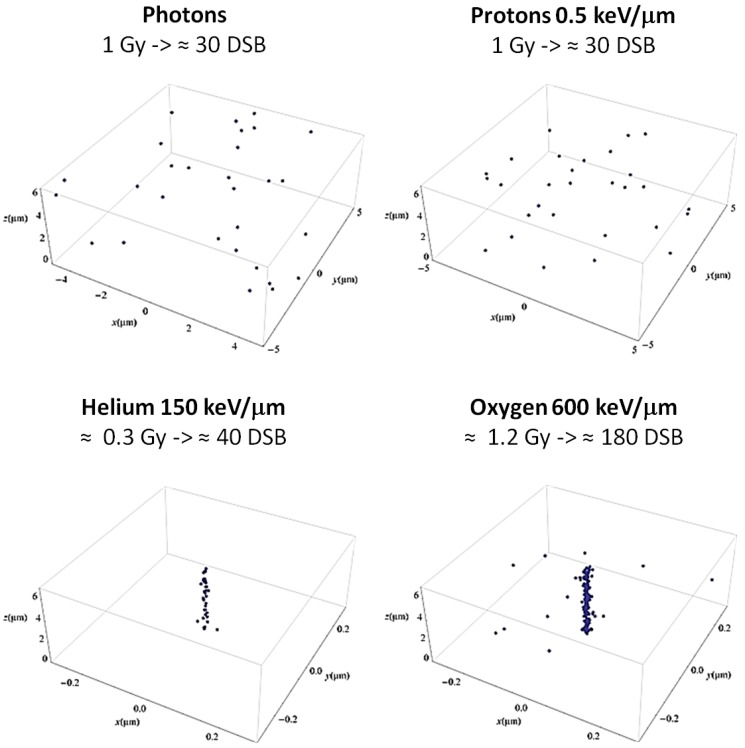
Simulated patterns of DSB distribution after photon and ion irradiation in a typical cell nucleus (radius of ≈5 μm). Protons are shown at the typical LET assumed in the entrance channel, resulting in a photon-like distribution of lesions (sparsely ionizing radiation). Low energy alpha particles and oxygen ions are also shown as representative of the typical target fragments produced in PT. In this case, a considerable dose is released in the nucleus after a single particle traversal, and a high dose is released close to the particle track, resulting in the induction of an increased number of close-by DSB (densely ionizing radiation). The DSB distributions were calculated with the Local Effect Model (LEM) [14,15], where an amorphous track structure model is adopted.

Apart from the advantages offered by the peculiar depth-dose profile, protons also show an enhanced biological effectiveness in cell killing [4]. This is related to the increased Linear Energy Transfer (LET) compared to X-rays when protons are close to the Bragg peak. High LET values are associated with a localized energy deposition resulting in the induction of enhanced, unrepairable biological damage [5]. Consequently, charged particles are often defined as densely ionizing radiation, in contrast to photons being considered sparsely ionizing radiation (see Figure 1 for a comparison of expected Double Strand Breaks [DSB] distributions after low and high LET irradiation). The increased effectiveness of charged particles compared to photons is quantified by the Relative Biological Effectiveness (RBE), defined as the ratio of photon and charged particle doses resulting in the same biological effect. The RBE is a complex quantity, depending on physical parameters (*i.e.*, particle type, dose, LET) as well as on biological ones (*i.e.*, tissue type, cell cycle phase, oxygenation level, end point). In a clinical context, RBE is used to calculate depth-dose profiles in terms of biological dose (or RBE-weighted dose, *i.e.*, physical dose × RBE), thus representing a key parameter to compare PT to X-rays for prescribed doses.

**Figure 2:**
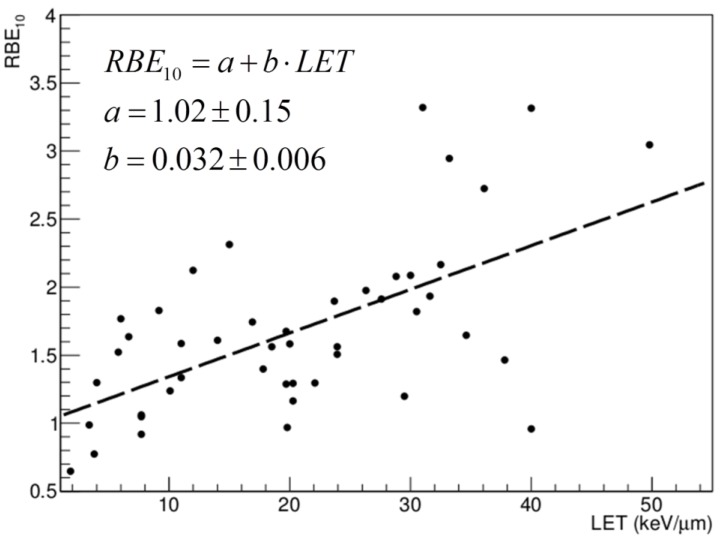
RBE values for 10% survival, as extracted from PIDE [7]. The dashed line shows the tendency to an increase in RBE with LET and is the result of a linear fit. All values extracted from the database were pooled together, independent of α/β ratio. This is allowed when looking at RBE for 10% survival; the consistency of this approach was checked by separate fit analysis (not shown). Interestingly, the fit parameters are in line with those presented by Paganetti in his recent review [6]. In that case, when looking at a restricted range of dose-averaged LET<15keV/μm, the linear fit produced RBE_2Gy_ = 1.02 + 0.052xLET_d_ and RBE_6Gy_ = 0.99 + 0.042xLET_d_.

Nevertheless, despite the comparably large experience in the therapeutic use of protons, there is still much that has to be elucidated concerning the biological response elicited in cells and tissues by protons compared to photons. This is reflected especially in the large uncertainties that can be found in the literature concerning the definition of an RBE–LET relation for protons [4,6]. In Figure 2 a collection of RBE values for 10% survival as extracted from the Particle Irradiation Data Ensemble (PIDE [7]) is shown. Large fluctuations are observed in published data sets for the description of RBE in tumor cells as well as in surrounding healthy tissues. Such fluctuations are in general larger for *in vitro* compared to *in vivo* experiments, the former representing the majority of available data. In most of the cases cell killing as measured by means of clonogenic survival assay is the end point studied with *in vitro* experiments. This information then represents the basis for the evaluation of Tumor Control Probability (TCP) in treatment planning. However, cell killing alone might not be representative of side effects eventually induced in normal cells, which are likely to be part of a more complex biological response. Thus, other *in vivo* end points (e.g., lung fibrosis, spinal cord injury) might be more appropriate for the calculation of Normal Tissue Complication Probability (NTCP). Furthermore, the majority of *in vitro* measurements have been performed with V79 cells, having a low α/β ratio (α and β parameters defined according to the Linear-Quadratic parameterization [8]), while *in vivo* experiments were performed by looking at early-reacting tissues, having in general a high α/β ratio [9]. Thus, since RBE is also dependent on α/β ratio, a direct quantitative comparison of *in vitro vs. in vivo* outcomes might be misleading. At the same time, it is now clear that cell killing alone is a simplistic end point to describe tumor response. This is due to the impact of the tumor microenvironment on the response, communication, and interaction among different cancer cells and among healthy and cancer cells, as well as among targeted and non-targeted cells [10].

Even though a comparably large amount of data is available nowadays, the large uncertainties and the many aspects to be taken into account when pooling them together (e.g., different experimental protocols, different biophysical models, *etc.*) hinder the definition of a simple and unique RBE–LET relationship that could be easily adopted in treatment planning. Thus, a fixed RBE equal to 1.1, based in particular on *in vivo* results, is currently adopted in PT in order to describe the increased effectiveness of proton radiation compared to therapeutic photons [4,6]. This fixed RBE is assigned to protons over the whole range. Indeed, despite the large fluctuations observed, several studies support the idea that RBE = 1.1 seems to be a reasonable approximation, and at this time there is no clear and unique clinical evidence against this assumption [4,6]. In other words, even if this assumption is notoriously wrong, it does not necessarily modify the clinical responses.

Considering the described background, discussion has arisen recently in the community concerning the need for a better understanding of radiobiological aspects related to PT. In fact, it is now well established that significant clinical improvements could still be obtained, provided that an increased and more accurate knowledge of the cell and tissue response to proton radiation is achieved. On the one hand, this would allow for increasing the efficacy of PT both in tumor killing and sparing of normal tissues; on the other hand, it would be helpful to identify clinical cases that would clearly benefit from using protons instead of X-rays. Obviously, a central aspect of the discussion is whether the use of a fixed RBE in PT is still appropriate, or whether current knowledge justifies a switch toward a variable RBE, taking into account the dependency on LET, tissue properties, dose, and dose fractionation. We will discuss how results obtained in studies on aspects of clinical relevance, for instance biological range extension and RBE variations due to target fragmentation, point to the latter. In addition, in this regard the data that have appeared in the literature in the last few years suggest that the biological response induced by protons differs for several end-points from that obtained after photon irradiation. This is true especially at the tissue level for clinically relevant aspects—for instance, angiogenesis and cell migration—as well as for induction of secondary cancers [11,12]. More generally, evidence has also been reported showing that different patterns of gene expression can be associated with exposure to different radiation qualities, which might elicit different molecular mechanisms, especially concerning cell responses in terms of cell cycle regulation and DNA repair [12,13]. Taken together, those results are in favor of a new approach, according to which high-energy protons should no longer be considered as a photon-like low LET radiation (*i.e.*, similar pattern of induced damage at the nanometer scale) with the clear advantage of a superior tumor targeting. On the contrary, high energy protons and photons may have both distinct physical and biological properties, which likely deserve further investigation. This is sometimes referred to as the “new paradigm of proton radiobiology” [12].

In this review, the advances in the study of proton radiobiology will be summarized and discussed. Specifically, the differential biological response induced by protons compared to photon radiation will be considered at different levels of complexity, from the sub-cellular to the tissue level. We will then discuss the results obtained in recent studies on the biological range extension, suggesting the relevance of an accurate description of RBE variations at the distal end of the SOBP. This would allow fully exploiting protons’ depth-dose profile, while at the same time reducing the risk of side effects in nearby OAR. The impact of recoil target fragments on the overall RBE will also be considered, underlining the importance of further investigation in this direction in order to better elucidate the potential contribution of low energy secondary fragments, especially concerning effects induced in normal tissues along the entrance channel. Considering all those aspects, our conclusions would be in favor of a reevaluation of the current approach based on the use of a fixed RBE [16,17,18].

## 2. Radiation Quality-Dependent Cell Response

In order to summarize the current knowledge in terms of cell response induced by different radiation qualities, we will describe the modulation of biological effects due to proton compared to photon radiation at different scales, from the sub-cellular to the tissue-organism level. A special focus will be given to aspects of more direct clinical relevance.

### 2.1. Sub-Cellular Level: DNA and Non-DNA Targets

Differences in the biological response induced by protons compared to photons were reported in the last decades, especially concerning the analysis of DNA damage induction and processing. DNA DSB induction by different radiation qualities shows that, even though similar patterns of initial induced DSB are produced by γ-rays and protons, clear differences emerge when looking at the rejoining process. In fact, a correlation can be observed between the enhanced fraction of remaining DSB as measured 2 h after proton irradiation and a gradually increased LET, resulting in RBE values significantly above 1.0 [19]. Evidence can be found in the literature about increased induction of DSB as well as higher numbers of more complex Clustered Lesions (CL) induced by protons in plasmid DNA in comparison to γ-rays [20,21,22]. Similar data were obtained by irradiating melanoma cells at different SOBP positions (plateau, peak, and distal end regions) [20]. Furthermore, it is often proposed that simple and complex lesions might be processed by different repair pathways, as for instance by Non-Homologous End Joining (NHEJ) and by Homologous Recombination (HR), respectively. There are hints that lesions induced by proton irradiation might be preferentially repaired by HR, thus reflecting an increasing complexity [23]. Since HR is a much slower repair mechanism compared to NHEJ, this would affect the number of residual lesions measured late after irradiation.

Differences in the patterns of radiation-induced DNA damage markers were also shown with modern immunostaining techniques. Even though contradictory data are reported in relation to an increased number of induced γH2AX foci after high-energy proton irradiation [24,25,26], there seems to be a general agreement on the fact that a larger foci size can be attributed to protons, which is considered a marker of enhanced lesion complexity [24,27,28,29]. Associated with the DNA molecule are also epigenetic changes related to radiation exposure. In this regard, experiments have shown that while exposure to X-rays is usually associated with hypo-methylation, proton radiation produces hyper-methylated DNA, both in normal and cancer cells [30]. While hypo-methylation is generally considered a factor leading to genome instability, the effects of hyper-methylation are still debated [31]. However, the different response in terms of epigenetic modulation is of interest for the potential impact on secondary cancer risk evaluation, and thus deserves further investigation.

The biological effects induced by ionizing radiation are known to be partially mediated by the production of free radicals, in particular by the so-called Reactive Oxygen Species (ROS). It is also recognized and tested that increasing ionization densities result in increased production of ROS [32,33,34]. For instance, it has been shown that 250 MeV protons at the end of the range lead to more rapid ROS increase compared to X-rays [34]. In addition, it has recently been hypothesized that differences in the production of ROS might be the reason for the enhanced effectiveness in inactivating cancer stem cells as observed for protons compared to photon radiation [35]. While ROS-mediated DNA damage has been a topic of research for decades, attention is recently increasing on the role of ROS in the induction of damage to cellular membranes, which are now considered as important non-DNA targets [36,37,38,39]. Some evidence showing that proton irradiation produces massive membrane damage was reported [40,41], but studies directly comparing effects at membrane levels induced by proton and photon radiation are currently missing.

Still at the sub-cellular level, but not directly related to DNA, are the differences in the gene expression patterns after exposure to different radiation qualities, which were recently observed by different groups. A recent study revealed that there is a clear difference in gene expression patterns induced by different radiation species in human bronchial epithelial cells [13], which can be related to distinct ionization densities, while not being strongly dependent on the physical dose delivered. A differential expression of genes related to apoptosis signaling, cell cycle control, and DNA damage response [24], as well as genes related to angiogenesis and inflammation response [11], seem to be dependent on radiation quality. The idea that different radiation qualities induce different DNA damage patterns has been for many years one of the recognized explanations for the increased effectiveness of charged particles compared to photons. However, the recent evidence reported above is supportive of the concept that cell response is influenced by the radiation quality at different levels of complexity, including the molecular one. This is why, even though there are not many studies dealing with radiation quality, radiogenomics appears to be a promising field of research [42]. Particularly intriguing seems to be the possibility of combining data on local tumor control with molecular data, aiming at the identification of biomarkers for radiation response, which could contribute to predicting tumor response to therapy [43].

### 2.2. Cellular Level

Radiation effects induced at the cellular level include some of the classical well studied end points, for instance cell killing, apoptosis, and cell cycle perturbations. Concerning the first, many *in vitro* studies are available, as performed by means of the standard technique of cell colony forming assay. Many studies have been performed with rodent cells, but studies on human cells are also found [44]. Specifically, V79 cells, characterized by a low α/β ratio, were the most adopted cell line. These studies are useful to show the dependence of cell killing on LET (see also Figure 2), and have been reviewed in detail [4,6,20,44,45]. Overall, an average RBE of ≈1.2 can be associated with a depth corresponding to the middle of the SOBP (mid-SOBP). When focusing on data obtained with human cell lines, having in general a higher α/β ratio, the average RBE is reduced to ≈1.1 [9]. Interesting studies can be found in the literature, showing the tendency toward an increase of RBE when investigating the survival of normal and cancer human cell lines exposed to protons at increasing LET [46]. At the same time, it is well known that an enhanced efficiency in the induction of cell inactivation can be measured at different positions along the SOBP [47,48]. This was confirmed recently by the combined experimental and modeling study published by Chaudary *et al.* [17], where normal and cancer human cell lines were employed (AG01522 and U87, respectively). However, considering the large uncertainties affecting the determination of RBE values, one has to keep in mind as a general rule that these uncertainties reflect in the final accuracy of treatment planning, whereas clinical settings require that error is below 3.5% in terms of biological delivered dose [16,49].

Several attempts have been made to model the RBE as a function of parameters of clinical interest (e.g., LET, dose fractionation, α/β parameters). Also in this context, one problem is represented by the large uncertainties affecting the experimental evaluation of the parameters of interest. The fact that RBE models rely on databases that are limited in terms of end-points considered represents an additional drawback. However, several models have been developed and tested against experimental databases of cell inactivation RBE [6,50,51,52,53,54,55,56]. An analysis of such models is beyond the scope of this review. Even though these models have not yet found clinical application, they are useful to highlight the RBE dependence on LET. For instance, a trend of a linear increase of RBE with LET was obtained, as well as a pronounced dependence on α/β parameters.

The choice of an RBE equal to 1.1, currently adopted in PT, largely depends on the data discussed, and is therefore considered of great relevance. The main limitation to the use of RBE values as measured by means of cell inactivation is that, even though they are expected to give reasonable predictions in terms of TCP, they are not likely to be fully appropriate for the description of NTCP. For instance, a cell might not be able to replicate anymore, but still be active from a metabolic point of view, thus influencing tissue response through interactions with surrounding cells.

These considerations lead us to the discussion of radiation-induced apoptosis. It is well known that apoptosis mechanisms can mediate the effectiveness of radiation effects, and that different cells/tissues can show different levels of apoptotic response. Generally speaking, most tumors do not undergo apoptosis [57], while in normal tissue apoptosis is much more common. The effectiveness of proton radiation in inducing apoptosis has been investigated in the past. In general, a tendency toward an increased apoptotic response is observed for increasing LET [32,58,59], which can be a concern for NTCP in OAR exposed in the high-LET region. Evidence has been reported that photon and proton radiation induce apoptosis through different signaling mechanisms [24,32,33,60], suggesting that some apoptosis-resistant tumors may be triggered in alternative apoptosis pathways by protons. In some cases, a different modulation of genes involved in the apoptotic response is observed (e.g., GZMP, SPP1, BCLG); in other cases, proton irradiation results in a significantly higher activation of genes involved in the pro-apoptotic response (e.g., p38, JNK, MAP, Bax, p21), in contrast to the activation of pro-survival signaling after γ-ray irradiation (e.g., ERK). This is considered to be supportive of the hypothesis mentioned above, *i.e.* that different radiation qualities elicit a different multi-scale biological responses [12]. Similar conclusions can be obtained when considering cell cycle perturbations. However, a differential response in terms of cell cycle changes between proton and photon radiation is not always observed [26,59,61,62]. At the same time, it was shown that cells can react with different expressions of genes to control cell cycle progression after proton and γ-ray irradiation, the first leading to upregulation of cell cycle stimulator genes (e.g., KRAS, Cyclin F), the second resulting in upregulated cell cycle blocker genes (e.g., CDKN2A) [24].

### 2.3. Tissue Level

Here we will focus on the analysis of tissue end-points having clear relevance in PT treatments: angiogenesis, inflammation modulation, invasion and migration effects, tumor recurrence, and secondary cancer induction.

It is well known that angiogenesis is an important aspect of tumor progression [63]. It is also well established that photon radiation stimulates angiogenesis [64,65,66,67,68], and in clinical practice anti-angiogenic factors are administered to patients in order to increase treatment efficacy [69]. On the contrary, some evidence has been reported indicating that densely ionizing radiation induces an anti-angiogenic response [70]. With regards to proton irradiation, low LET protons downregulate pro-angiogenic factors, as for instance VEGF, IL-8, IL-6, HIF-1α, in both *in vitro* and *in vivo* experiments [11]. Additionally, *in vitro* experiments also show that proton irradiation can significantly inhibit formation of new blood vessels, without inducing alterations in the morphology of already existent ones [71]. Taken together, these results suggest that proton irradiation can negatively modulate angiogenesis, by the parallel processes of down-regulation of pro-angiogenic factors, and inhibition of neovascularization.

The inflammation response resulting from exposure to ionizing radiation is an important aspect in PT applications, especially concerning the induction of normal tissue complications. At the same time, due to the complex network behind the inflammatory response, inflammation at the tissue level is a difficult effect to predict based only on information at the sub-cellular and/or cellular level. Some data in terms of post-therapy inflammation are available from clinical studies, where patients treated with photons plus protons and with protons only were compared [72]. A significantly higher expression of proteins involved in the inflammatory response was observed in patients belonging to the first category, with 62% of them developing serious negative pulmonary changes (scored as 2 or greater). In contrast, none of the patients treated only with protons showed such serious side effects, while 67% of them had no injury at all. However, when the end-point is the inflammatory response, the comparison might be easily biased by the superior tumor targeting (and normal tissue sparing) in patients receiving only proton therapy. At the same time, limited and controversial data can be found in the literature, coming from studies conducted *in vitro* as well as with animal models [11,73,74]. Thus, even though a general tendency to a reduced inflammatory response after proton irradiation is observed, further studies are needed in order to support and confirm these preliminary indications. *In vivo* studies would be most welcome. Importantly, a further motivation to additional investigation comes from the fact that the study of the inflammatory response is currently considered a promising end point for prediction of NTCP [6].

The picture appears to be clearer concerning the modulation of invasion and migration effects as induced by protons. A lower level of migration and a reduced invasion potential has been reported after proton irradiation in comparison to X-rays [11]. Protons can elicit a biological response, which includes anti-invasive and anti-migration behavior, and signaling mechanisms play a key role in this context [75,76,77].

In light of the discussion above, it is reasonable to expect that photon and proton radiation differentially modulate non-targeted effects [10]. In fact, the possibility that biological effects are also induced in cells not receiving radiation would imply that the actual target extends beyond the irradiated field. The inflammatory response and apoptotic signaling seem to be involved in such effects [78,79,80,81]. The immune system plays a central role in this context. Thus, new opportunities might arise from an adequate combination of PT and immunotherapy [82,83,84].

Concerning tumor recurrence, there are clinical data showing that cancer relapse is likely to take place in regions that for whatever reason did not receive enough dose or were not treated at all, while a very low rate of recurrence is reported in patients correctly receiving the prescribed dose [85,86,87]. Sethi *et al.* recently reported that no correlation was found between recurrence rate and tumor regions where protons’ LET was lower than expected, stressing at the same time the importance of an accurate dose delivery according to the treatment plan [88]. Similarly, there are no randomized trials available today to compare secondary cancer induction in patients treated with proton *vs.* photon radiation. The limited clinical data present in the literature suggest that the rate of secondary cancer induction is about one-half after proton compared to photon radiation therapy [89,90,91]. Epidemiological data in pediatric patients exposed to protons support the view that a reduced risk is expected with this treatment compared to X-rays [90,92].

## 3. Range Uncertainties: Biological Range Extension and the Role of RBE

The main motivation behind the development and spreading of PT relies on the superior tumor targeting offered by the Bragg peak depth-dose profile. Assuming that the same dose is delivered to the tumor, this allows a significant sparing of normal tissues in comparison to conventional radiotherapy treatments. However, in clinical practice some limitations to the full exploitation of the advantage offered by protons arise, as the consequence of physical and biological uncertainties relative to the actual particle range. The risk of inducing serious side effects can be associated with the steep dose gradient characterizing the distal end of the Bragg peak. This is true especially for those tumors that might be treated with a single field, having an OAR located close to the distal end of the SOBP. In fact, the actual position of the Bragg peak inside the patient is always affected by uncertainty, which can be on the order of 3% of the expected range [93]. Extra margins are thus defined in order to account for these uncertainties [94,95]. However, in clinical practice this is a reason to avoid using single fields pointing in the direction of critical organs behind the SOBP [96]. In fact, multiple fields minimize the influence of range uncertainties. However, at the same time they result in a larger radiologic path length to the target, thus increasing the fraction of normal tissues receiving radiation. This is the typical case when PT is adopted to treat prostate cancers, where sparing the rectum is considered to be critical in treatment planning.

From the physics point of view, range straggling associated with proton–matter interaction contributes to the final uncertainty being higher for high energies and deep-seated tumors. Additional sources of uncertainty are imaging artifacts [97,98], issues in the conversion of CT Hounsfield units to proton stopping power [99,100], anatomical changes, and sub-optimal positioning of the patient during treatment [101]. Consequently, apart from defining safety margins, different technical strategies have been developed in order to get information about the actual proton range obtained inside the patients [102].

**Figure 3:**
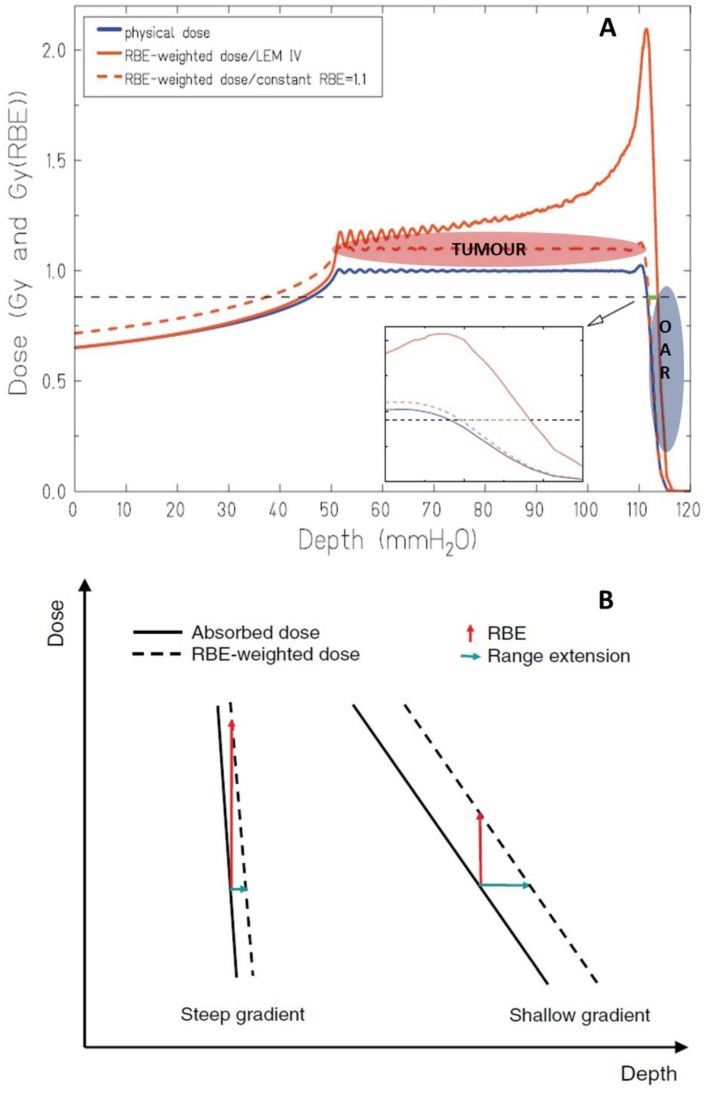
In panel (**A**) physical and RBE-weighted dose are shown, as obtained with a constant and with a variable RBE. Special emphasis is given to the differences at the distal fall-off, where OAR might be located. In panel (**B**) the different impact in terms of range extension that can be expected when comparing steep and shallow dose gradients is shown. Adapted from Grün *et al.* [103].

In addition to the physical uncertainties, biology can contribute with the so-called “biological range extension” [55,64,103]. This is a consequence of the parallel increase of LET and decrease of dose taking place at the distal fall-off of the SOBP, resulting in an increase of RBE. This effect can result in a significant biological dose in normal tissues beyond the tumor region, thus introducing additional uncertainty (Figure 3A). According to the fixed RBE of 1.1 adopted in PT, this biological range extension due to a gradually increasing RBE is currently not directly considered in treatment planning. The role of a variable RBE in the estimation of the biological range extension has been recently studied using a Monte Carlo- [55] or a LEM-based [103] approach.

A pronounced dependence on dose and α/β parameters is reported in both studies. Despite some quantitative and qualitative differences attributed to the different biophysical models employed, the largest extensions are obtained for the combination of low doses and low α/β ratios, when a large increase in RBE is expected. The biological extension does not seem to be dependent on target volume, but is rather strongly influenced by the width of the distal penumbra, generally becoming more pronounced at higher energies [103]. As summarized by Figure 3B, in general steep and shallow dose gradients will correspond to short and large range extension, respectively. Importantly, the dose gradient that can be associated with the distal penumbra is also strongly dependent on the beam modulation techniques. Even though specific cases deserve dedicated analysis, in general active scanning will produce steeper dose gradients compared to passive modulation systems for irradiation of the same target, as a consequence of the lower beam energies needed in the first case. The biological extension can span up to about 4 mm, thus is on the same order of magnitude as the size of critical structures usually encountered in radiotherapy close to the treated volume (e.g., optical nerve, pituitary gland, *etc.*).

Apart from these two studies, few data more directly related to a clinical context can be found in the literature concerning the evaluation of the impact of the biological extension. In the treatment of hypopharyngeal carcinoma, the effective dose deposited in the spinal cord increases by a factor of 1.5 when considering a variable RBE instead of a fixed one [56,104]. MRI data concerning the direct evaluation of the biologically effective range in patients treated with PT for tumors in the lumbar region show an average overshoot of 1.9 mm in the lumbar spine [105].

We conclude that the biological range extension cannot be neglected in proton treatment planning. The biological extension seems to be particularly important in cases where the proton beam traverses very heterogeneous tissues, considering the strong dependence of the biological extension on α/β parameters. This aspect becomes even more relevant since delivery uncertainties are being reduced due to technical advances [55,95]. This means that safety margins might be reduced and OAR might be more exposed to range extension resulting from biological factors.

## 4. Nuclear Interactions

In their interaction with the traversed biological tissues, protons (and charged particles in general) mainly lose energy by means of electromagnetic Coulomb interactions with target electrons. The rate of energy loss increases with depth as particles slow down, and this is reflected in the gradual increase in LET, finally resulting in the Bragg peak profile. In addition, nuclear interactions can take place with the atomic nuclei of target material, which can be subdivided into elastic and inelastic collisions. The outcome of the inelastic interactions is the production of a spectrum of secondary target fragments, depending on the characteristics of the primary ion beam and the target material. These particles can assume different values of energy and LET. Therefore, it is not a simple issue to determine their contribution to the overall RBE.

The *abrasion-ablation model* is usually adopted to describe fragmentation processes of interest in particle therapy [106,107]. The abrasion and ablation steps refer to the particle removal during ion–ion interaction and to the subsequent nuclear de-excitation, respectively. Depending on whether the collision between projectile and target nuclei is peripheral or central, we can expect mass removal or total destruction of the nuclei to take place. Due to geometrical reasons, peripheral collisions are normally prevalent.

The total reaction cross section is a key parameter for the description of nucleus–nucleus collisions. In fact, it is used to calculate the probability of reactions taking place between a projectile and a target nucleus. Starting with an initial number of particles *N_0_* traversing a slab having thickness *x*, we can calculate the expected number of particles at the exit window as follows:

(1) $$N(x)=N_{0}\cdot e^{-x/\text{λ}}$$

where λ represents the mean free path for a given total reaction cross section σ*_R_*, and is expressed as:

(2) $$\text{λ}=\frac{M_{mol}}{N_{A}{\text{ρσ}}_{R}}$$

In Equation (2) we also have the dependence on the characteristics of target material, with *M_mol_* indicating the molecular mass and ρ the density of the target, respectively; N_A_ is the Avogadro constant. Due to their importance in nuclear physics, the reaction cross sections have been measured intensively in the last decades, and parameterizations are also available. Specifically, the Bradt–Peters equation is considered a good representation of the observed total reaction cross section [108,109,110]. Several variants of the equation can be found, but in order to represent the general case we can report it as follows:

(3) $${\text{σ}}_{R}=\text{π}r_{0}^{2}c_{1}(E){\left[A_{p}^{1/3}+A_{t}^{1/3}-c_{2}(E)\right]}^{2}$$

where *A_p_* and *A_t_* indicate the atomic mass number of projectile and target, respectively; and *r_0_*, *c_1_*, and *c_2_* are parameters, depending on the specific parameterization adopted. This expression indicates that, for a given projectile, we can expect an increased cross section (*i.e.*, increased fragment production) for increasing *A_t_*. For high-energy protons, Equation (3) becomes [111]:

(4) $${\text{σ}}_{R}\approx 53\cdot A_{t}^{2/3}\;\text{mb}$$

In water, *N*σ*_R_* =0.012 cm^−1^ and the mean free path is λ = 82 cm. Therefore, approximately 1% of the protons in the therapeutic beam experience nuclear interactions in a cm of tissue, corresponding to approximately 20% for a deep tumor site. However, different materials will strongly affect the resulting fragment spectra. An example of clinical relevance could be the presence of bone tissue in the radiation field before the tumor. Indications concerning the expected average energy of recoil fragments can be obtained with the so-called Goldhaber formula. In the case of proton beams at 180 MeV, which is a typical energy for a therapeutic beam, it can be expressed as follows [112]:

(5) $$E_{F}=\left[\frac{A-F}{A-1}\right]\frac{3}{5}\frac{p_{F}^{2}}{2m_{0}}\text{MeV}$$

where *A* and *F* indicate the target and fragment mass, respectively, *m_0_* the proton rest mass, and *p_F_* the Fermi momentum. The dependence of *p_F_* on the fragment mass can be approximated as [113]:

(6) $$p_{F}=281\cdot (1-F^{-0.568})$$

Thus, the Goldhaber formula indicates that, for a given target material, the average fragment energy will be higher for light fragments. At the same time, we can expect more energy transferred to the fragments for increasing target mass.

**Figure 4:**
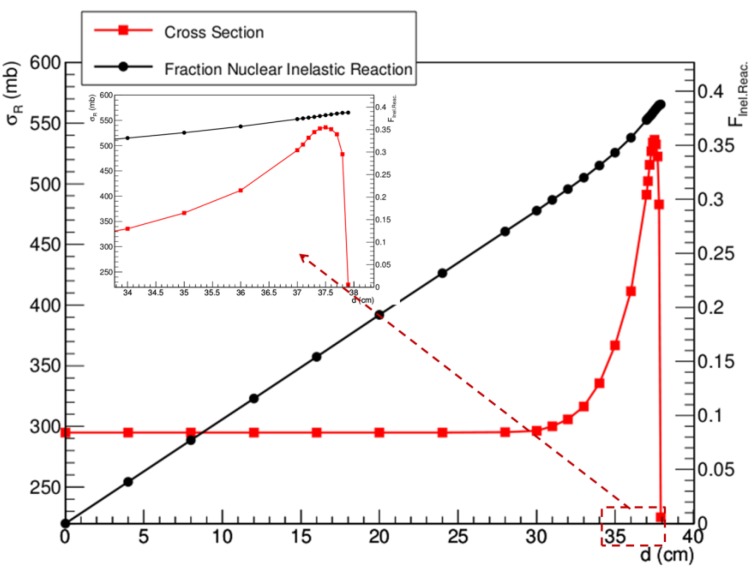
Fraction of primary protons undergoing inelastic nuclear reactions along the Bragg peak in a water target for 250 MeV initial beam energy (black symbols). The corresponding total reaction cross section values at the given positions were calculated according to the database currently adopted in the treatment planning system TRiP98 [115,116] (red symbols). The inset shows a zoom in the last 4 cm of range.

**Figure 5:**
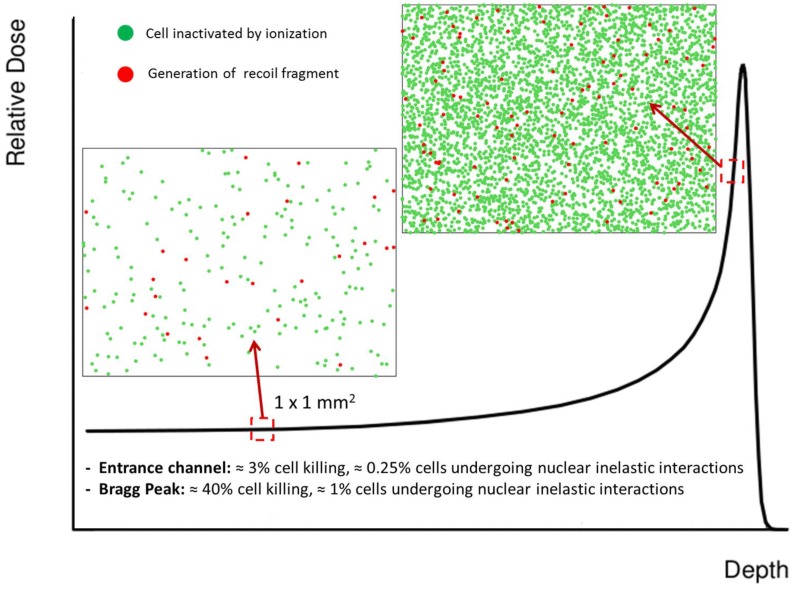
The figure schematically shows the impact of ionization and target fragmentation in tissue sections of 1 × 1 mm^2^. The effect is considered at two different positions along the depth-dose profile. The LEM code was used to estimate cell survival probability [14,15]. The expected contribution of target fragments is calculated assuming that in water about 1% of primary protons undergo nuclear inelastic interactions per traversed cm. We chose a fluence of 2.5 × 10^8^ p/cm^2^ for the primary beam, and assumed that cell nuclei cover an area of ≈100 μm^2^. This means that we can expect an average of 250 particles traversing a nucleus. When following protons traversing 1 mm of tissue (stack of 100 cells), we thus expect an average of 0.25 protons undergoing inelastic nuclear reactions. In other words, we will have one fragmentation event every fourth stack of cells, which translates to what is shown in the figure. Importantly, we only show cells where target fragments are generated, but a fraction of those fragments might have enough energy to traverse a few cell nuclei before coming to rest. This is true especially for light fragments (see also Table 1). Even though both the contributions of ionization and fragmentation increase when approaching the Bragg peak, at that position the biological effect is mainly due to ionization events. On the contrary, in the entrance channel the predicted survival is high, and therefore a significant role might be played by low-energy target fragments.

Target fragmentation can be associated with several aspects of potential clinical relevance [114]. In fact, secondary fragments obviously contribute to the overall dose deposited in the patient; this is reflected in a distortion of the SOBP if not compensated for in the treatment plan. Moreover, target fragments characterized by low energy and/or high atomic number can be expected to be associated with an enhanced RBE. Finally, nuclear interactions can also lead to the production of neutrons, eventually depositing the dose outside the planned target volume. In Figure 4 we plot the relative fraction of primary particles at given depths in water for a proton beam having an initial energy of 250 MeV. Calculations were performed according to Equations (1) and (2), and the corresponding total reaction cross sections are also shown. It is clear that a significant fraction of primary particles is lost due to inelastic collisions and that only about 60% of them reach the Bragg peak region. At the same time, it is apparent that the reaction cross section has an almost constant value in the entrance channel, but it shows a pronounced increase in the last 8–10 cm of range. This behavior might be considered when implementing accurate treatment planning software including target fragmentation effects. In Figure 5 we use a schematic approach to give indications concerning the expected contributions of electromagnetic interaction and fragmentation along the Bragg peak in terms of biological effects. Remarkably, if we assume as first-order approximation that each cell hit by secondary fragments will die, our calculations suggest that about 10% of the biological effect induced in the entrance channel might be associated with target fragments. At the same time, due to the slowing down of primary protons, this contribution is reduced to about 2% when approaching the Bragg peak. In Table 1 we summarize the average expected physical parameters associated with target fragments for a 180 MeV proton beam in water, according to Equation (5). Interestingly, it appears that light fragments might have enough energy to hit more than a single cell.

**Table 1:** Expected average physical parameters for target fragments produced in water by a 180 MeV proton beam. The initial average energies of secondary fragments are calculated according to the Goldhaber formula (Equation (5)), considering the dependence of *p_F_* on the fragment mass (Equation (6)).

Fragment	E (MeV)	LET (keV/μm)	Range (μm)
^15^O	1.0	983	2.3
^15^N	1.0	925	2.5
^14^N	2.0	1137	3.6
^13^C	3.0	951	5.4
^12^C	3.8	912	6.2
^11^C	4.6	878	7.0
^10^B	5.4	643	9.9
^8^Be	6.4	400	15.7
^6^Li	6.8	215	26.7
^4^He	6.0	77	48.5
^3^He	4.7	89	38.8
^2^H	2.5	14	68.9

### 4.1. Transport Codes

From the above discussion it emerges that a correct assessment of reaction cross sections is particularly important considering the diffusion in the last decade of Monte Carlo (MC) codes for medical applications. In fact, these codes make use of reaction cross sections as input parameters in order to calculate the probability of nuclear interactions taking place when charged particles traverse matter. MC tools allow detailed simulation of nuclear interactions in complex clinical setups (e.g., 3D geometry, multiple materials) and they are more flexible compared to analytical tools. Yet MC simulations can be very CPU- and time-consuming, and this still hinders their use in the clinics. Any uncertainty affecting the estimation of reaction cross sections propagates in the calculations and thus affects the accuracy of final results. This obviously represents a critical point when thinking of potential clinical applications. For instance, an estimation of the biological impact of target fragmentation will strongly depend on the accurate prediction of the expected fragment spectra, seeing as LET (and therefore RBE) are dependent on particle charge and energy. Among the MC codes adopted in medical physics research, GEANT4, FLUKA, and PHITS are probably the models of largest interest for charged particle therapy. Differences among the codes arise from the different nuclear models adopted to calculate reaction cross sections, as well as from different computation algorithms. The comparison of the models used in the 3 MC codes for different projectile-target combinations [117] points to significant differences in the predicted cross sections, notwithstanding the small differences among the nuclear models. For protons hitting a water target, this is particularly evident for energies below 100 MeV, which are of clear relevance in therapy. Unfortunately, not many experimental data are available at these energies to be compared to predicted values. Similar differences are observed in the comparison of FLUKA and GEANT4 for carbon-ion therapy [118]. The results show that even for the same MC code the final outcome can strongly depend on the nuclear model adopted to describe nuclear interactions (*i.e.*, BIC LI *vs.* G4QMD for GEANT4). Moreover, depending on the physical quantity considered, the predictions of the two models can present differences of some tens of percent. The authors also suggest the direct coupling of MC codes with radiobiological modeling aiming at an accurate estimation of biological effects due to nuclear interactions. According to the above discussion of the potential role attributed to target fragmentation, this conclusion can be extended to proton therapy as well. There is a need for additional experimental cross-sections in therapy-like conditions for benchmarking of MC codes. This would represent a crucial step toward clinical applications of MC-based treatment planning in charged particle therapy.

### 4.2. Target Fragments and RBE

**Figure 6:**
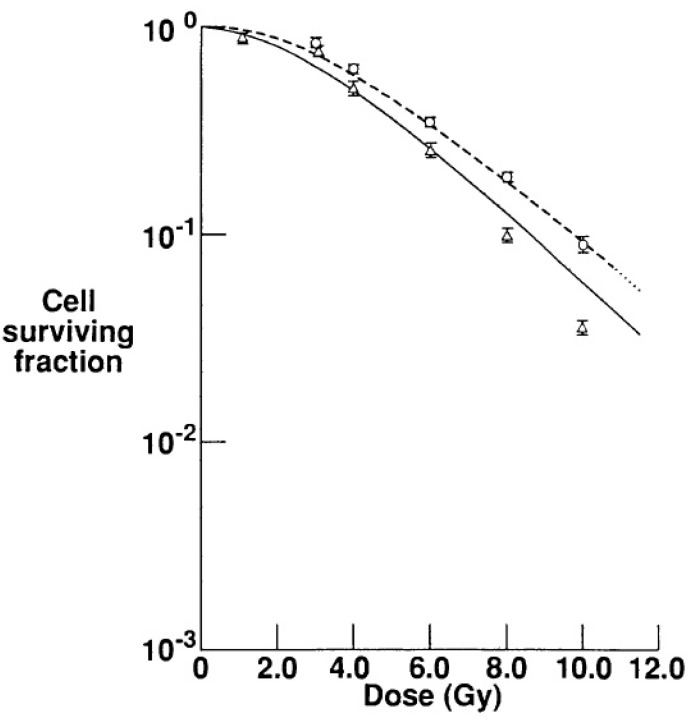
Experimental data of cell inactivation obtained with CHO cells after irradiation with ^60^Co γ-rays (circles) and with 160 MeV protons (triangles) are shown, together with modeling analysis. The fitted curves show that γ-ray data are well described by the model when nuclear reactions are not considered (dashed line), while a good fit is obtained for proton data by including fragmentation processes in the model (solid line). Adapted from Cucinotta *et al.* [120].

The fact that a potentially significant contribution to the deposited dose could be due to target fragments has been known for decades [119]. Indications concerning the contribution to the RBE in the entrance channel associated with recoil fragments after high energy proton irradiation were reported in 1991 by Cucinotta *et al.* [120]. The biophysical model developed for that study allowed switching nuclear interactions *on* and *off* in the calculation algorithm. Thus, they applied the model to fit survival data obtained with V79 cells after irradiation with γ-rays and with 160 MeV protons. As shown in Figure 6, almost identical results are obtained when γ-ray data are fitted with the photon model and with the proton model without including nuclear interactions. This suggests that, due to the low LET of high-energy protons, the two radiation qualities are similar for the end point of cell inactivation. At the same time, proton data are well fitted by the model when the production of secondary fragments is considered, indicating a significant contribution coming from low-energy recoil particles. The authors suggest in their conclusions that in a clinical context this contribution might be especially relevant at the low doses deposited in the entrance region because of the high RBE values expected for the secondary particles.

**Figure 7:**
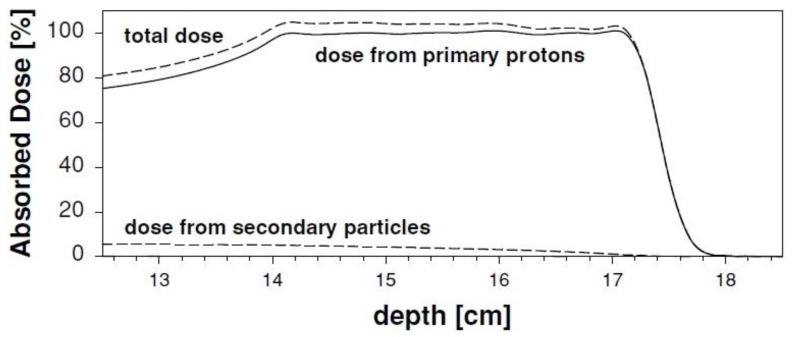
The total absorbed dose along a proton SOBP is shown, by separating contributions coming from primary protons and from secondary fragments (A < 5). Adapted from Paganetti [114].

Monte Carlo simulations can be used to calculate the contribution of target fragmentation to the delivered dose. For instance, Paganetti [114] used an unmodulated 160 MeV proton beam and a SOBP of 3 × 3 × 3 cm^3^ in water. The analysis was restricted to secondary particles with an atomic number lower than 5. RBE was investigated for three different cell lines, and the amorphous track structure model by Katz and co-workers was adopted for the simulations [121,122], similar to the model used by Cucinotta *et al.* [120]. This study shows that a small but significant contribution to the physical dose deposited in the entrance channel comes from secondary particles, and that it gradually decreases along the SOBP (Figure 7). Concerning the biological dose, an increase in RBE up to ≈8% at 2 Gy was predicted. This is expected to depend also on the specific end point considered (in this case, inactivation of V79 cells). The author concludes that secondary protons and alpha particles should be taken into account for an accurate estimation of the biological dose [114]. Importantly, considering the high RBE involved, the biological effects induced in the entrance channel will have a more pronounced dependence on dose compared to the primary beam. Obviously, the α/β ratio of tissues in the entrance region is also expected to have some role. The effects of neutrons produced by nuclear reactions were also considered. Specifically, secondary neutrons were found to be responsible for an enhanced RBE in the distal fall-off due to the production of protons. Minor contributions to the biological dose distal to the SOBP were found to be associated with neutrons (<0.04% of target dose). However, the author concludes that final predictions in terms of late cancer induction risk associated with neutrons should also take into account the background particles originating from the beam delivery system, and therefore deserve further investigation.

In a more recent study [18], the production of target fragments was simulated both in water phantoms and in patients (prostate and chordoma). All secondary particles were included in the analysis. Specifically, light particles (A < 5) were scored and tracked down to zero kinetic energy, while heavy secondaries (A > 4) were assumed to deposit their energy locally, so their residual range would be below the grid size. Thus, the authors were able to show that when considering the contribution of secondary protons to the dose-average LET, a significant impact is expected in the delivered biological dose. This is true especially concerning the plateau region and the lateral penumbra. Furthermore, the energy spectra were calculated and shown for the most abundant secondary particles (α-particles, O, C, N, BE, B). From the analysis of the spectra, it is evident that a clear contribution to RBE can be associated with α-particles. Even though the picture is less clear for heavier particles, it is likely that such fragments also play a role. However, an explicit RBE model should be included in the analysis in order to quantify the contribution of each radiation quality. Importantly, these effects are expected to take place in healthy tissues, thus resulting in an increased risk of late side effects, including cancer.

From the studies considered above, it emerges that target fragmentation might be an important aspect to consider for a correct description of NTCP in the entrance channel. This is true for protons but also for charged particle therapy in general. However, in this regard very limited information is available in the literature in terms of biological experiments. This is partly due to the technical complexity in performing dedicated experiments. In fact, experimental determination of the RBE due to target fragmentation is not a simple issue.

Even though direct indications could be obtained only by means of biological experiments, it is obviously not possible to disentangle the effects due to secondary fragments from the ones due to the primary ion beam. However, some information can be extracted from published studies focusing on the analysis of biological effects induced at different positions along the whole radiation field. For instance, according to the low LET and high energy of the primary beam, an RBE close to 1.0 would be expected in the entrance channel. However, a small but significant increase is observed in experiments for the RBE in the entrance region as well. This increase is usually in the order of 10%–40%, depending on several factors including the specific ion specie employed and the biological end point considered. For instance, RBE values between ≈1.05 and ≈1.18 in the entrance channel were reported by analyzing the surviving fraction after irradiation of CHO cells with a 65 MeV modulated proton beam [48]. Similarly, RBEs equal to 1.16 ± 0.04 and 1.10 ± 0.03 were obtained by Guelette *et al.* when looking at the regeneration of intestinal crypts for mice irradiated in the initial plateau with an unmodulated and modulated 200 MeV proton beam, respectively [123]. Other interesting results come from studies performed with carbon ions. Remarkably, RBE > 1.0 was measured by Ando *et al.* when considering both tumor growth delay and skin reactions in mice irradiated with beams accelerated at different energies [124]. RBE values of 1.33 ± 0.02/1.42 ± 0.02 and of 2.97 ± 0.05/5.04 ± 0.08 were reported by Karger *et al.* as resulting from the analysis of D50-values for radiation tolerance of rat spinal cords after carbon ion irradiation with 6/8 fractions at plateau and at peak, respectively [125].

Because of the fluctuations and the discrepancies observed in the results of different experiments, and due to an incomplete understanding of the underlying processes, empirical solutions are currently adopted in treatment planning in order to take into account the effects due to the dose deposited by the recoil fragments (for instance, the enhanced RBE = 1.1 assumed for protons along the whole range). However, until a more detailed knowledge of these effects is available, this approach could result in an under- or overestimation of the RBE in the entrance region, thus affecting the quality of the delivered plan.

Therefore, an improvement in the understanding of the biological role that can be attributed to target fragmentation after charged particle irradiation is expected to have practical implications and to be beneficial in the therapeutic context. A more accurate description of the RBE in the entrance region would allow for better definition of the peak-to-entrance RBE ratio, which largely characterizes the therapeutic advantage of ion beam therapy. At the same time, it would be relevant for the evaluation of side effects that can take place in the irradiated healthy tissues in front of the tumor, and that also show a dependence on the radiation quality adopted for the treatment. An appropriate definition of the risk of side effects is also critical when evaluating the balance among the TCP and the NTCP. In this context, a deeper understanding of the RBE systematics related to irradiation with different radiation qualities, will help the clinicians to adopt improved therapeutic decisions under different circumstances. At the same time, the deposition of dose associated with target fragmentation, even though low if considered in absolute terms, could be relevant concerning the risk assessment for secondary cancer induction. This is true in particular considering the high LET associated with those fragments. This aspect represents an important topic of discussion not only in radiation therapy, but also in the context of space research as a consequence of astronauts being exposed to galactic cosmic rays, consisting of accelerated charged particles in the range from protons to iron ions.

In this framework, it clearly emerges that further studies aimed at elucidating the contribution of target fragments to the overall RBE would be welcome. Experimental as well as theoretical efforts would be justified in order to quantify the increase of RBE that can be expected in different beam and target configurations. This step is needed in order to estimate the significance of the expected RBE increase, and eventually implementing countermeasures.

Importantly, while for heavier charged particles the picture is further complicated by the onset of projectile fragmentation, protons represent an ideal configuration for the analysis of target fragmentation since no projectile fragmentation is expected to take place. Thus, a better understanding of the problem in PT could then be of profit for charged particle therapy in general.

## 5. Conclusions: The Times They Are a-Changin

The recent results obtained in different fields of research and summarized in this review indicate that the complexity contained in the RBE parameter might be too drastically underestimated by the choice of a fixed RBE, as currently adopted in PT. RBE = 1.1 still sounds reasonable when considering the information resulting from the large number of *in vitro* and *in vivo* experiments. Moreover, it is correct to state that no clinical data are available at the moment reporting induction of serious side effects and clearly against the use of a fixed RBE. However, like a dog chasing its tail, this is in part the consequence of the use of the fixed RBE itself. In fact, because of awareness of the problem, the distal edge of the SOBP is rarely positioned close to OAR, thus limiting the risk of serious side effects. Disregarding RBE variations might lead to bias in favor of protons compared to photons, while the treatment plan calculated by taking into account RBE variations actually results in the delivery of a dose lower than expected to the tumor and higher than expected to normal tissues [126]. It is now also recognized that an RBE obtained from inactivation data can be only partially representative of effects other than the killing of tumor cells.

Another fact that should be considered when thinking of possible improvements in PT is that protons generally produce more heterogeneous dose distributions compared to photons. On the one hand, when discussing the possibility of biological optimization we have to keep in mind that the experiments on which RBE calculations are based were performed with homogeneous dose distributions. On the other hand, we have to consider that this aspect could influence in a different way the effects induced in tumor and in normal tissues. The comparison among different treatment plans (e.g., protons *vs.* photons) is thus made on the basis of the same biological dose deposited in the target, while different doses can be deposited in healthy tissues. The radiation field obtained with protons in the entrance region might be even more heterogeneous when target fragmentation is included in the plans. Importantly, it is also likely that only a fraction of the total volume of OAR receives a given dose. Thus, it is not easy to extrapolate the risk that can be attributed to the whole organ. A possible solution to these problems might be represented by supporting the introduction of biological models with the consideration of the effects on a voxel by voxel basis instead of on the level of entire organs [6,127]. This seems to be a relevant approach for an accurate calculation of NTCP.

We conclude that the efforts needed to increase the accuracy of the evaluation of RBE in PT, both in the normal tissue and along the SOBP, are well justified. A final goal of these efforts should be the implementation of biological models in PT treatment planning. However, this is likely to lead to problems in comparing data from different centers, if different models are used. PTCOG and ICRU may reconsider the issue of a constant RBE and suggest a model to be adopted in different centers.
